# Barriers and Facilitators to the Implementation of an Integrated Youth Services Network in Ontario

**DOI:** 10.5334/ijic.6737

**Published:** 2022-12-14

**Authors:** Debbie Chiodo, Stephanie Lu, Thepikka Varatharajan, Jean Costello, Brian Rush, J. L. Henderson

**Affiliations:** 1 Youth Wellness Hubs Ontario, CA; 2 Centre for Addiction and Mental Health, CA; 3 Western University, CA; 4 Homewood Research Institute, CA; 5 School of Public Health Sciences, University of Waterloo, CA; 6 University of Toronto, CA

**Keywords:** integrated youth services, youth mental health, barriers, facilitators, developmental evaluation, implementation science

## Abstract

**Introduction:**

In response to the challenges of the traditional mental health system for youth both in Canada and abroad, models of integrated youth services (IYS) that span the integration of mental health, health, substance use, eucation, employment, peer support, and navigation into ‘one-stop shops’ are being established nationally and internationally. IYS models, however, need to be better described and evaluated to inform the replicability of this approach in other jurisdictions.

**Description:**

This paper describes the implementation of an IYS in a small urban city and rural county in Ontario, Canada, including insights from key informants into barriers, facilitators, and lessons learned.

**Discussion:**

This evaluation identified a number of barriers and facilitators to the implementation of the IYS model in this specific context. Implementation facilitators included youth and family engagement, network partner collaboration, leadership, governance structure, community enthusiasm and support, and collaborative funding models. Barriers to implementation included the COVID-19 pandemic and related public health restrictions, the diverse needs of youth, change management, sustainable funding, and transportation.

**Lessons learned:**

By establishing a shared vision of delivering youth services across the integrated network, and engaging youth early in the process of model development, IYS have the potential to transform the service system for youth and their families. Meeting the diverse needs and challenges of youth who live in rural or small urban communities will enhance service delivery and experience for young people.

## Introduction

### Background

Mental health problems in youth are a growing concern in Canada, and elsewhere. Studies suggest that mental and substance use disorders commonly begin in adolescence and early adulthood, causing significant distress and impairment [1,2]. In Canada, about 1 in 5 youth have at least one mental health disorder [3,4,5] with up to 70% of these disorders persisting into adulthood [1]. If left untreated, mental health problems are associated with negative outcomes that endure well into adulthood [6].

Despite a number of evidence-based treatment and support options being available, youth face a number of barriers to high quality treatment [7,8,9,10]. Globally, there is consensus among clinicians and researchers in child and youth development and mental health that current service delivery systems focused on youth are failing to meet the challenging and complex needs of youth and their families [11,12,13]. Barriers to access to services for youth include fragmented or siloed services with disruptive transitions for youth and their families; long wait lists; a lack of developmentally and culturally appropriate services; age restrictions at critical transition periods; over-emphasis on illness-based and medical models; and services that fail to consider the preferences, perspectives, and voices of youth and families [12,13,14,15,16].

### Problem Statement

In response to the significant challenges of traditional mental health systems for youth both in Canada and abroad, models of integrated youth services (IYS) that span the integration of mental health, health, substance use, education, employment, peer support, and navigation into ‘one-stop shops’ are being established nationally and internationally. These IYS are designed, typically in collaboration with youth, to bring traditionally separate services (e.g., mental health, substance use, health, housing, education, etc.) together into one community-based setting to provide comprehensive services for youth and young adults. Leaders in the international IYS space include *headspace* centres and Orygen Youth Health in Australia, Jigsaw in Ireland, Forward Thinking Birmingham in the United Kingdom, and youth One Stop Shops in New Zealand [13,15,16]. In Canada, system transformation for youth services is being led by Foundry BC (https://Foundrybc.ca), ACCESS Open Minds (https://accessopenminds.ca), Youth Wellness Hubs Ontario (www.youthhubs.ca) [12], and Aire Ouverte (www.quebec.ca). IYS is a promising practice that addresses youth needs more holistically by embedding youth and family voice in all aspects of service design and delivery [12]. Given the holistic nature of care provided within IYS, the bioecological model is a theoretical model that has been proposed to align with the development of IYS models. The bioecological model examines individual development within multiple systems of influence as well as through interactional processes between the individual and the environment [17]. The major components of IYS models have been developed to align and compliment the basic tenets of the bioecological model with the understanding of the dynamic nature of multiple system influences and interactions within a youth’s service journey [18]. Researchers have identified that IYS models need to be better described and evaluated within their community context to inform replicability in other jurisdictions [e.g., 15]. This paper describes the implementation of an IYS in a small urban city and rural county in Ontario, Canada, including insights from key informants into barriers, facilitators, and lessons learned.

## Integrated Youth Services Network: The Grove-Youth Wellness Hub Ontario

The Integrated Youth Services Network (IYSN) was established in 2018 in two regions in Southwestern Ontario: Wellington County (a rural setting) and the city of Guelph (an urban setting). The local population includes over 46, 000 youth between the ages of 12 and 26 years who live in Wellington County and Guelph, representing one-fifth of the total population. Almost a third of the youth live in the city of Guelph. The Grove Wellington- Guelph (formally the IYSN) was developed to address important gaps in the continuum of services and access challenges for youth between the ages of 12 and 26 years, with a focus on meeting the needs of rural and transitional aged youth in Wellington County and the city of Guelph. As with most other regions in Canada, youth in Wellington County and Guelph have significant need for mental health and substance use supports; however, the challenges faced by rural youth due to isolation, transportation, and stigma from community members may necessitate different considerations when developing IYS.

Integrated governance is central to the development of the Grove Wellington-Guelph because meaningful collaboration between all members of the network to guide planning and service development, resource allocation, and accountability is critical to the overall success of the model [12]. For The Grove Wellington-Guelph hub sites, integrated governance consisted of a consortium of community service organizations which formed a Partnership Table, a Youth Advisory Committee, and a Family Working Group. During the early phases of the network, the Partnership Table included representation from nine mental health and community-based organizations invested in the initiative, including youth and their families. The Partnership Table is responsible for overall governance of the network, including external communication and partnership development through quarterly community update meetings. The Partnership Table also named the IYSN, *The Grove Hubs*.

The network also developed a strategy around youth and family engagement early in the development by encouraging each hub site to have its own Youth Action Council. It was during these Youth Action Council meetings that service partners would invite a designer to work with youth to discuss their desired look and feel of the hub sites. During these discussions youth also had the opportunity to share what they believed was attainable for the space, potential barriers and how they might be mitigated, opportunities to receive mental health counselling, activities and games that the hub site could provide to promote overall wellness, and suggestions on making the hub site a welcoming space.

In an effort to address barriers and gaps within the service system for youth and families, The Grove Hubs planned to implement seven service sites throughout the region during its first five years. The Grove Hub has a $15 million campaign target that includes capital costs. The projected budget for each site was approximately $600,000. Securing funding for this budget included in-kind contributions of the IYSN service and community partners, fundraising dollars, and grant awards. At the time of this evaluation, The Grove Hubs Partnership Table had secured funding for the initial stages of planning of the network through significant community fundraising campaigns and operating grants, but sustainable funding for full implementation of the model had not been realized. In 2020, the Ontario Ministry of Health announced annualized base funding ($650,000/year) for ten IYS sites across the province (Youth Wellness Hubs Ontario (YWHO) sites, www.youthhubs.ca) as a commitment to their investment of improving the mental health and addiction system for young people. In 2021, The Ontario Ministry of Health expanded their investments of IYS to include an additional four communities, of which The Grove-YWHO was one. In the spring of 2021, the Grove Hubs officially became a YWHO site (renamed: The Grove–YWHO; www.youthhubs.ca).

YWHO is a provincially-funded, and philanthropically supported, initiative of 22 integrated youth service hubs in rural, northern, and culturally diverse Ontario communities [12]. The Grove-YWHO, like all other YWHO sites, focuses on meeting the needs of diverse youth (including rural and transitional-aged youth) by offering a range of comprehensive services in Wellington County and Guelph [19]. It provides rapid access to mental health, health, and substance use services integrated with other community and social support services (e.g., education, employment, housing, navigation), delivered in youth-friendly spaces for youth ages 12 to 25. The Grove-YWHO was co-created with youth and family members where youth and families were actively involved in hub governance, service design, and service delivery.

The Grove–YWHO model of integrated youth services adheres to the YWHO’s core components which include: 1) youth and family engagement; 2) integrated governance and partner collaboration; 3) accessibility; 4) inclusive and culturally diverse services that reflect the population groups; 5) integrated service delivery model; and 6) measurement-based care [12]. In addition to the implementation of core components, The Grove-YWHO is aligned with the Canadian federal, provincial, and territorial governments’ priorities to expand access to mental health and addiction services for youth, and to spread evidence-based models of community mental health care and culturally appropriate interventions for youth between the ages of 12 and 25. During the time of this project planning, the coronavirus disease 2019 (COVID-19) was rapidly spreading across the globe, disrupting The Grove-YWHO’s in-person service delivery for youth, and to some extent, progress on the development and implementation of the IYS model.

## Methods

### Study Context

In the fall of 2020, The Grove-YWHO partnered with a local research group (Homewood Research Institute; HRI; https://hriresearch.com) to conduct an evaluation of the local IYS model. The objectives of the evaluation included: 1) to support the development, implementation, and continuous improvement of the model; and 2) to provide initial evidence to inform the replicability of the model within other jurisdictions or contexts. To help guide the evaluation process, an evaluation subcommittee was established that included representation from The Grove-YWHO, local service providers, youths, and families.

### Ethics and Consent

This evaluation received ethics approval from the Community Research Ethics Office in Waterloo, Ontario (CREO-202). The purpose of the study was explained to all participants, and participation was voluntary. Written informed consent was obtained for participation.

### Study Design

This evaluation examined barriers and facilitators to the implementation of The Grove-YWHO model of integrated services with a qualitative methodology. A developmental approach [20] was taken since the service model was considered to be a complex intervention in a dynamic environment and within the early stages of implementation. Further, although the design of The Grove-YWHO service model largely adhered to the YWHO core components, its initiation, implementation, and delivery were unique to the Wellington County and Guelph communities. Thus, a developmental approach was also appropriate because it provided real-time data to inform decision-making as part of ongoing adaptation and implementation within this unique context.

### Data Collection

Data were collected from key-informant interviews with The Grove-YWHO site supervisors (N = 10) and program leads (N = 2). Site supervisors typically managed the day-to-day operations of the hub, while program leads often managed a particular hub program (e.g., mental health services). Site supervisors and program leads were invited to participate in one-hour virtual interview to discuss their roles and experiences in the implementation and delivery of the service model, including barriers, facilitators, and lessons learned. Semi-structured interview guides were developed to gather information on the implementation process, with particular focus on planning, engaging, and executing as outlined in the Consolidated Framework for Implementation Research (https://cfirguide.org).

### Data Analysis

The interviews were recorded, transcribed verbatim, and analyzed using a thematic approach [21]. First, author (SL) reviewed transcripts in full length to get a general impression of the data and then drafted a theme code set using inductive (i.e., themes that emerged from the transcripts) and deductive (i.e., themes used in the interview guide) reasoning [21,22]. This theme code set was verified by a second author (TV), before author (SL) applied it to all transcripts using qualitative data analysis software *NVivo 10* [22]. Using the theme code set, author (SL) compiled a summary of themes with supporting quotations and identifiers removed. These themes and the supporting quotations were presented to co- authors (TV, JC, BR) and the Evaluation Subcommittee to ensure that their interpretation was clear and as objective as possible. Together, the authors decided which supporting quotations best represented the identified themes for inclusion in the study.

## Results

The key informant interviews identified a number of barriers and facilitators to the implementation of The Grove-YWHO model. Facilitators of the model included youth and family engagement, network partner collaboration, leadership, integrated governance structure, community enthusiasm and support, and collaborative funding models. Barriers to the implementation of the model were the COVID-19 pandemic and related public health transitions; diversity of needs of youth; change management; securing sustainable funding; and transportation. Identified themes and supporting quotations from key informants are presented in Tables 1 and 2. Results as discussed more fulsomely in the discussion section.

**Table 1:** Identified facilitators to the implementation of the IYS model and sample quotations.


FACILITATORS	SAMPLE QUOTATIONS FROM KEY INFORMANTS

Youth and family engagement	“If any region is starting this they must engage with youth. What better people to go to than the people accessing services. The youth know the youth best.” “Our main slogan is ‘For youth by youth.’ It’s important to have youth involved in anything that’s going on and not just assume what they want.” “I understand why we tend to focus on the youth part, and not always families, so how do you make sure you get families onboard? What supports do they need and how do we make sure they are being met?”

Network partner collaboration	“Service providers are coming together and keeping their eye on the ball, instead of the silo mentality that we had in the past.” “We’re all there for the right reasons … there’s that shared understanding of the barriers for service in our area, and that shared goal of breaking down those barriers.” “It has been helpful being connected as a network … we’ve been able to share what works … we can help each other stretch and challenge each other to change. It has been such a great network of people who want to help.” “I’ve always felt that you could be honest about how you’re feeling. Everyone is very transparent … You do feel safe if you disagree about something … it’s okay to say that.”

Leadership	“[The Grove-YWHO Director] has been huge. Her ability to make things happen, to reach out, to raise money, her foresight about what she knows and what she doesn’t and when she needs to bring in somebody, her commitment to the ideals.” “[The Grove-YWHO Director] has single handedly moved this entire initiative forward with her own passion and tremendous productivity. Everybody who sits around the IYSN [Partnership] Table is in awe of her passion, commitment, and impact. So really, without that one individual driver, this would not be happening. I don’t say that lightly. I can’t imagine anybody having the vision, the drive, the persistence, to move this ahead, given all the various barriers to making it happen that are real.”

Integrated governance structure	“A lot of the [early] conversations from the Partnership Table were about not wanting to be a charity, taking away from other charities that we’re partnering with … having MOUs [memorandums of understanding] is a huge piece.” “We have guiding principles and we follow them. We try to go with the consensus model as opposed to a voting structure. We all are in it together to move the ship.”

Community enthusiasm and support	“The community momentum for this project is unbelievable. It’s really overwhelming at times and I think that speaks to the need. It is, especially with COVID, we know that the need is so great.”

Collaborative funding models	“I think they are doing an awesome job in setting up their fundraising … from the outset they did a really good job of trying to assess who needed what and in what areas.” “As people saw [The Grove-YWHO Director] getting grants and more money being raised, there was more excitement for people to continue to be engaged.”


**Table 2:** Identified barriers to the implementation of the IYS model and sample quotations.


BARRIERS	SAMPLE QUOTATIONS FROM KEY INFORMANTS

COVID-19 Pandemic	“The idea was to have all these different programs, have people talking about it. Having the school come for field trips. We had a great plan to make that happen, but then COVID … people are sick of being online, but our hands are tied. We know what we want to do, but it has just been dragging on for so long.” “We had tons [of youth] signed up for those initial in-person activities during the April break … spots were full, they wanted to come, but of course participation dwindled a bit when we went back to virtual. They are craving that in person stuff.” “The pandemic has certainly taken a lot of our energy. It has forced us to take a step back to think about how to survive this. But I appreciate the Director being so focused and driving it.” “COVID-19 has slowed the renovations quite a bit. Closures have stopped work. Renovations started in October and they are in the final stages now. Costs for materials like lumber have increased, which affected our fundraising needs.”

Diversity of youth needs	“While the majority of youth living in rural areas face challenges related to isolation and having fewer opportunities for development and socialization, some urban youth in need of programs and supports in Guelph face challenges related to homelessness, hunger, discrimination, etc.” “We have high rates of mental health and addictions issues in our community. There’s a lot of homeless people that hang around [The Hub] sometimes … We have nutrition programs that we run. A lot of kids will come to that and sometimes it’s the main meal for that day.” “In the rural hub sites there are concerns that stigma and gossip will be a barrier to youth participation”.

Change management	“Others were skeptical and I don’t think it’s because they did not believe that the model would work. I think it is change. I think change is hard for people. But people are getting their heads wrapped around it. We’re getting there.” “It’s a lot of work … that can be a barrier when we’re trying to run our own organizations, and especially when we’re in multi-area organizations, you know. I am already sitting on a lot of committees and meetings. But, I don’t know, it can be challenging to find the time. But again, because we believe in it so much, we figure it out, we get it done.”

Sustainable funding	“We know that this model and transforming the way we’re delivering services is going to cost us an enormous amount of money …although our fundraiser campaign is doing really well, the ongoing sustainable dollars will be a concern for us.” “I’m generally a believer that if you build a good thing the funding will come. However, in this era of scarcity, it’s really, really important that our advocacy with our provincial funders is strong…. at some point the government will have to start shifting gears from a pandemic response to a pandemic recovery, and that is both from the economic and social perspective.”

Transportation	“Transportation may present a challenge to some hub sites. For example, the YMCA of Three Rivers Guelph Y is 12 minutes from downtown Guelph by car, but 1 hour by bus. Attempts have been made to work with Guelph Transit, but it will take more than the Guelph hub site alone to make a compelling case for an added bus route. Another example is East Wellington Community Services in rural Erin. While the hub site has access to vehicles, they will be largely dependent on parents to drive youth as there are no local transportation options”.


## Discussion

### Facilitators to the implementation of an integrated youth service network

This study highlighted a number of facilitators to the implementation of an IYS: youth and family engagement, network partner collaboration, leadership, governance structure, community enthusiasm and support, and collaborative funding models. An essential core component of IYS models, which was also an underlying pillar of the Grove-YWHO, is the inclusion of meaningful youth and family engagement processes [11,12,14,15]. Within the Grove-YWHO, youth and families were involved in hub processes from the start and Youth and Family Engagement Committees actively met on a monthly basis to provide direction on youth space, desired services, equity and access, evaluation activities, and the development of programs and resources at the site. An evaluation of youth and family engagement within the Grove-YWHO suggests positive and valued experiences in many aspects of model development [19]. Youth participation in decision-making, leadership, site selection, service development, and evaluation is recognized as a best practice for ensuring high quality service to young people [23]. Meaningful youth engagement within IYS ensures that youth are involved as co-creators in services and processes that impact them [24]. Including families in the development of IYS models can have positive impacts on direct service outcomes for youth and their families [18]. The bioecologoical model emphasizes the critical role of social relationships and family/significant others in development. Many IYS models use a broad and flexible definition of family and their engagement strategies involve both natural families and significant others (e.g., support person). While this practice has been suggested to be expanded within other IYS models [e.g., 18], research on meaningful engagement of families within IYS models is needed.

For IYS to be successful, the models require collaboration between network partners involved in service design and delivery. IYS partnerships within the network require a considerable investment of resources (e.g., time, staffing), cross-sectoral collaboration, and a shared vision and approach in the application of IYS principles and standards. Historically, policy-related barriers among community organizations in youth mental health, such as arbitrary or rigid age limits for service provision, have stifled meaningful and sustained collaboration efforts [13,25,26]. The Grove-YWHO relies on significant partner collaboration to transform the way that services for youth and their families are planned, delivered, and evaluated in their communities. One of the guiding principles of partner collaboration for the Grove-YWHO is a shared vision among network partners in improving services for young people and their families.

Bringing together organizations and services into an IYS network requires the development of governance structures that promote coordination, collaboration, integration, and accountability [11,13]. The Grove-YWHO’s integrated governance structure consists of a network Partnership Table, youth engagement working group, and a family engagement work group. The network Partnership Table is responsible for overall governance of the network, and is comprised of representatives of all partnering organizations, core service providers, youth and families. Engagement at the governance level, both within the network, and across the various partnering sectors has enabled The Grove-YWHO to manage its deliverables, risks and processes in a successful manner. In addition, key informants described the commitment and leadership of the hub director as a critical factor to success.

### Barriers to the implementation of an integrated youth service network

This evaluation also highlighted a number of barriers to the implementation of The Grove-YWHO: the COVID-19 pandemic, diversity of needs of youth, change management, sustainable funding, and transportation. The COVID-19 pandemic has significantly impacted the lives of youth and their families and their access to mental health and substance use services, especially in vulnerable communities [27]. Like many other service organizations across the globe, The Grove-YWHO responded to the pandemic by shifting to online provision of services with some mixed models of care (i.e., blend of in-person and virtual care). Although online service delivery ensured continuity of care during this time, it is not without its limitations [27,28]. For The Grove-YWHO, service providers found challenges keeping youth engaged and interested in online services. From their perspective, youth prefer to participate in in-person programming and to spend time at the hub sites. Online programming at The Grove-YWHO generally resulted in a lower attendance than expected. Moreover, service providers themselves faced stressors related to fears associated with the pandemic and fatigue with the public health restrictions [19].

Addressing the diverse needs of youth and families in Wellington County and Guelph was identified as a barrier to implementation. For example, providers noted youth living in rural areas in Wellington County face challenges related to isolation, and having fewer opportunities for development and socialization. On the other hand, youth living in the city experience significant poverty and food security challenges. As part of their commitment to health equity and understanding the diverse needs of youth and families in their community, The Grove-YWHO conducted an organizational assessment (i.e., Health Equity Impact Assessment, HEIA) of unintended health impacts (positive or negative) that its services and processes might have on vulnerable or marginalized groups of youth [19]. The Grove-YWHO identified a lack of culturally-specific services available that reflect the diversity of youth in the region. The Grove-YWHO currently has a robust equity, diversity, and inclusion plan for each of its sites to ensure that youth from diverse backgrounds feel welcome, safe, and represented when engaging with the hub and its staff.

Establishing an IYS is complex and dynamic, influenced by political, economic, and social factors [13]. A change of culture and practice is required at the clinical, managerial, and organizational levels. It is not surprising that key informants identified the need for adequate change management strategies to enable successful implementation and sustainability. An integrated model requires carefully developed partnerships, financial structures, and technological platforms for long-term sustainability of these highly integrated services [29]. Currently, evidence to guide managing the people side of the change required to implement models of IYS has not been well-articulated. Future studies should examine the processes, practices, and deliberate activities that may facilitate an integrated network, and its partners to move from its present state to a desired future state of a collaborative treatment and support model. Key informants also identified their concerns related to sustainable funding to support the hub long term. Despite base annualized funding from the government and an active fundraising campaign, key informants of the Grove-YWHO noted that ongoing advocacy to the government for additional funding is needed.

Finally, transportation has long been recognized as a barrier for young people’s access to services, especially for youth in rural and remote areas [30]. It takes youth and their family members considerable time, planning, and resources to get to the services they need. For The Grove-YWHO, transportation was identified as a barrier for some youth to access services in their community due to insufficient local public transit options. Finding creative ways to increase mobility for young people to access services such as offering free or discounted transportation resources (e.g., IYS vehicles, public transit, taxi) or developing virtual care innovations may help to address barriers and improve access for all youth and their families.

## Lessons Learned

In addition to the barriers and facilitators to implementation, key lessons learned were also uncovered from interviews with key informants and are described below. To some extent, these lessons are a direct reflection of the barriers and facilitators described above; however, we highlight four that emerged as particularly important and relevant for those seeking to implement an IYS in their own community.

1. *Engage youth early in the process*. Youth engagement empowers young people to be valuable partners in decision-making about factors that affect them personally and that they believe to be important. Youth engagement is an active and ongoing process that embeds the voice of diverse youth and representation at all levels of service planning, implementation and evaluation activities. Key informants noted that engaging youth from the start keeps the process honest, and recognizes that youth and families know best about what the services they need. Less is known about how to meaningfully engage families within IYS models, and future research should examine how to engage and empower youth and families in the process from the beginning and key considerations for their involvement.
2. *Understand who is accessing services and who is not*. Key informants noted that it is critical to have an understanding of the communities accessing services at the site to better understand and meet the needs of all youth. Asking questions such as “who is not coming and why are they not coming” will ensure that communities are actively finding ways to ensure that all youth have access to equitable, developmentally appropriate, timely service. Related, a major component to the bioecological model is understanding the temporal qualities of an individual’s development. IYS models consider service needs across development, and support youth holistically as they move into adulthood. Because youth accessing services within IYS can move in and out of service seamlessly over time as their needs change, youth are supported at important developmental transition points.
3. *A shared vision among network partners is important*. Developing partnerships and relationships take time and are necessary for effective service delivery. Key informants expressed the need to engage partners very early in the process, and create a common vision and roadmap for transforming services in the community. Participants also noted that an understanding of what each partner in the network needs to be able to work collaboratively is key. Since partnerships within IYS require involvement that cross sectors and organizations, adapting policies that are conducive to building relationships and that create incentives for collaboration is essential for IYS success [18].
4. *Expect a demanding process for change*. Key informants described the significant change management processes that was needed to establish the model and recognizing the time and commitment required from all partners was not an easy process.

## Conclusions

The experiences presented in this study serve to further our understanding of the barriers and facilitators to the implementation of IYS models in small urban city and a rural community. The Grove-YWHO is one example of a global movement to address system fragmentation and barriers to high quality care for youth and their families. IYS models provide comprehensive supports that emphasize early intervention and holistic care in youth-friendly physical spaces. Literature reviews of IYS models highlight the many different approaches and processes that have been implemented [9,10,11,12,13,15,16]. Recognizing that there is a need for a better understanding regarding the standards or core components of IYS models, future research is required to identify best practices to inform policy, research, and practice. In particular, future research that addresses the diverse needs of youth from different contexts (i.e., rural, small urban cities) accessing services within IYS is needed.

## Limitations

This paper described the implementation of an IYS in a small urban and rural context and provides insights into barriers, facilitators, and lessons learned. Models of IYS in Canada have developed fairly recently and data on the implementation of these models is limited. Because The Grove-YWHO is part of a scale-up of IYS across the province of Ontario (www.youthhubs.ca), we believe these findings can help to inform the replicability of the model for other jurisdictions in this province, and beyond. However, it is limited in its generalizability due to the local service context of The Grove-YWHO region. The small number of interviews (N = 12) may have impacted information power, which is an indicator of the adequacy of the information contained in the sample that is relevant for the study’s objective [31]. In addition, because only supervisors and program leads were interviewed for this part of the study, what are identified as barriers for youth is based on the interpretation of the supervisors and program leads, rather than the youth themselves who may or may not be able to access the hub. We also did not collect demographic information from participants such as years of experience working in the sector, which may have influenced their perceptions of the barriers and facilitators to the model.
